# Systematic review of communication technologies to promote access and engagement of young people with diabetes into healthcare

**DOI:** 10.1186/1472-6823-11-1

**Published:** 2011-01-06

**Authors:** Paul Sutcliffe, Steven Martin, Jackie Sturt, John Powell, Frances Griffiths, Ann Adams, Jeremy Dale

**Affiliations:** 1 Health Sciences Research Institute, University of Warwick, Coventry, CV4 7AL, United Kingdom

## Abstract

**Background:**

Research has investigated whether communication technologies (e.g. mobile telephony, forums, email) can be used to transfer digital information between healthcare professionals and young people who live with diabetes. The systematic review evaluates the effectiveness and impact of these technologies on communication.

**Methods:**

Nine electronic databases were searched. Technologies were described and a narrative synthesis of all studies was undertaken.

**Results:**

Of 20,925 publications identified, 19 met the inclusion criteria, with 18 technologies assessed. Five categories of communication technologies were identified: video-and tele-conferencing (n = 2); mobile telephony (n = 3); telephone support (n = 3); novel electronic communication devices for transferring clinical information (n = 10); and web-based discussion boards (n = 1). Ten studies showed a positive improvement in HbA1c following the intervention with four studies reporting detrimental increases in HbA1c levels. In fifteen studies communication technologies increased the frequency of contact between patient and healthcare professional. Findings were inconsistent of an association between improvements in HbA1c and increased contact. Limited evidence was available concerning behavioural and care coordination outcomes, although improvement in quality of life, patient-caregiver interaction, self-care and metabolic transmission were reported for some communication technologies.

**Conclusions:**

The breadth of study design and types of technologies reported make the magnitude of benefit and their effects on health difficult to determine. While communication technologies may increase the frequency of contact between patient and health care professional, it remains unclear whether this results in improved outcomes and is often the basis of the intervention itself. Further research is needed to explore the effectiveness and cost effectiveness of increasing the use of communication technologies between young people and healthcare professionals.

## Background

Maintaining an effective communication structure is essential for efficient interaction among care providers, patients and their caregivers [1]. Developments in the internet and mobile telephony are creating diverse approaches to achieving such interaction. The evidence base for their effectiveness and impact is limited. The current review evaluates communication technologies between patient and healthcare professionals within the context of young people who live with diabetes. Such technologies allow transfer of digital information between separate geographic locations, using physical or 'wireless' connections, for example: social networking sites (e.g., Facebook, MySpace); mobile telephony; Voice over Internet Protocol (VoIP) system (e.g. Skype); forums; email; short message service (SMS); multi-media message service (MMS); and N3 communications. Although these technologies are subject to change as new technologies are developed, the common feature is that they all allow remote access to a service (e.g. practitioner, nurse and specialist) and provide a means of supporting the provision of healthcare self management, education, communication and support. The review explores the comparative effects of different communication technologies, active interventions whose delivery may be facilitated by technology and technology-enabled remote interaction between health professional and patient, including passive monitoring with biofeedback or two-way dialogical communication or tailored information and support.

A growing number of young people experiencing concerns about their health may seek support from the internet and social network sites [2,3] but it remains unclear how their experiences of these sites affect their communication with health professionals, health behaviours and everyday living. UK data show approximately 90% of 16- to 24-year-olds had used the internet within the last three months and 70% report daily use (only 4% had never used it) [4]. Households with children are more likely to have access to the internet, especially if they are teenagers [5].

Childhood type 1 diabetes is a potentially life-threatening condition which is diagnosed in 15,000 children and young people under the age of 15 each year, and the total number is predicted to rise to 160,000 by 2020 across Europe [6]. Although type 2 diabetes is still less common than type 1 diabetes in children, the frequency of type 2 diabetes appears to be increasing [7]. Difficulties with controlling diabetes among adolescents and young adults are common [8], resulting in increased risks of long-term complications [9]. Poor control of diabetes has been related to the changes during puberty [10], problems of treatment compliance [11] and attendance at outpatient visits [12], reflecting a range of physiological, psychological and social factors. Several key priorities in the care of young adults with diabetes have been proposed [13]. First, to develop a strong relationship that will ensure continued follow-up to promote change in self-care behaviour. Second, to work in partnership with the patient to establish treatment goals that will foster a sense of success, self-efficacy, and engagement in self-care. Third, to ensure that high-risk adolescents with psychological problems have continuity of psychological care into the young-adult period. During the transition between adolescence and adulthood, maturational changes and life-long routines of self-care are frequently established. Communication technologies may provide several opportunities for supporting younger people to improve the frequency of contact and their relationship with healthcare professionals, thus supporting the transition of care between adolescent and adulthood.

A review of the effectiveness of psychological interventions on glycaemic control and psychological status found no statistical association between HbA1c and duration of follow-up (p = .275) or duration of therapy (p = .488) [14]. However, an association was found between improvements in HbA1c and an increased number of sessions (p = .001). Communication technologies therefore have the potential to increase contact between patients and health care professionals.

Recent reviews [15,16] evaluated interventions promoting information and communication technologies (ICTs) with health professionals and patients with chronic disease, but none have looked specifically at diabetes and young people. A significant effect on continuous behavioural outcomes and a non-significant effect on binary behavioural outcomes have been found [15]. In contrast, further research identified very limited evidence on the effectiveness of interventions promoting the adoption of ICTs by healthcare professionals [16]. While patients may find ICTs useful, healthcare professionals appear to be more resistant to adopting new means of communicating with their patients. This suggests that successful implementation is likely to require careful consideration and action to address the barriers to the adoption of these types of technology.

The review will assess the effectiveness and impact of technology-supported packages versus usual packages for the healthcare needs, support and education of young people with diabetes. We have chosen to focus on diabetes as it appears to be a transferable model for care of many chronic diseases [17]. Unlike previous reviews [15,16] we cover a specific age range, condition and communications technologies between patient and health professional and do not restrict the type of study included. It had the following specific aims: a) to describe the types of communication technologies available; b) to present the evidence for the effectiveness (RCTs) and impact (non-RCTs) on clinical, behavioural, psycho-social and care coordination outcomes; c) to describe the personal, family, educational, health service, broader societal costs and benefits associated with communication technologies for meeting healthcare needs for young people with diabetes; and d) to explore the theoretical underpinning of communication technologies with specific consideration of frequency of contact.

We utilised a *pathway of action *to understand the working of communication technologies in the diabetes healthcare context [15] coupled with several forms of communication technologies [18,19] or other self-management interventions [20]. Health communication technologies may act by combining information with additional services (peer support, decision support, behaviour change support) to allow interpretation of the information and internalisation; a combination of knowledge and enhanced self-efficacy with motivation enable users to change their health behaviours, leading to changes in clinical outcomes [15]. Social cognitive theory states that health behaviours are influenced by self-efficacy, or the belief in one's ability to perform actions that will influence outcomes [21], which, in turn, is influenced by goal setting and social support [22,23]. This can lead to changes in knowledge for improved health or health behaviours, affective parameters and self-efficacy. The combination of enhanced self-efficacy with motivation and knowledge may enable adolescents and young adults to change their health behaviours, which in turn, may change some clinical outcomes (e.g. HbA1c).

## Methods

### Search strategy

The search aimed to identify all references to diabetes and communication technologies. The search was undertaken in May 2009. The searches were intentionally broad to capture evidence related to all long-term conditions in young people. We did not restrict our searches to literature concerned with diabetes alone until the full paper sift, and the evidence identified for other long-term conditions will be reported separately.

A comprehensive review using standard methods of electronic bibliographic database searching with subject headings; key terms and words as well as hand searches of key journals, were undertaken following the general principles recommended in PRISMA guidance [24]. Specific strategies defined by review author(s) and assisted by an information specialist were developed. Electronic databases were searched including; MEDLINE; EMBASE; ASSIA; Sociological abstract; Social Studies abstract; PsycINFO; Cochrane Database of Systematic Reviews; Dissertation Abstracts; and Current Controlled Trials http://www.controlled-trials.com. Established public and personal networks were utilised to identify 'grey' literature. A broader web search was undertaken to assess more general information related to communication technologies.

### Search terms

A combination of free-text and thesaurus terms were used. 'Technology' search terms were combined with "Communication" and "Population" search terms (see Table 1 for details).

**Table 1:** Search strategy

*'Technology' search terms:* telemedicine; remote consultation; telecare; ehealth; e-health; e-learning; elearning; reminder system$; online system; interactive health communication; computer communication network$; communication aid; interdisciplinary communication; mobile phone; social network; facebook; myspace; virtual world; short messaging service; virtual clinic$; online clinic$; on-line clinic$; internet; world wide web; interactive health; computer assisted therapy; information technology; electronic communication$; digital divide; e-mail; email; telehealth
*"Communication" search terms:* health behaviour; health education; patient education health care delivery; adolescent health; health care system; health knowledge, attitudes, practices/; attitude to health; child health care; self efficacy; social support; health promotion; self care; attitude to computers; physician-patient relations

*"Population" search terms:* child$; teen$; paediatr$; pediatr$; boy$; girl$; youth$; schoolchild$; school child$; kid$; adoles$; minors$; under ag$; juvenile$; pubescen$; secondary school$; high school$; peer group$; highschool$; schoolage; school age$; young adult$; young person$; young people; student$; sixth form$; higher education; further education; undergraduate$; college student$; university student$; universities/; college$

### Inclusion and Exclusion criteria

#### Types of Studies

All types of study design published in all languages from January 1990 to May 2009 were included except letters, book chapters, commentaries and reviews.

#### Participants

Young people and adolescents with diabetes (type 1 or 2) based in the community, primary care, inpatient and outpatient populations were included. Studies that had a mean/median age below 25 years were included.

#### Intervention

All forms of communication technologies which involve a form of communication between patient and clinician, these include: social networking sites; mobile telephony; video-and tele-conferencing; forums; email; short messaging service, electronic monitoring. Interfaces which only involve parent and child, or peer-to-peer fall outside the remit of this review. Interventions involving the transmission of data alone without feedback were excluded.

#### Condition/disease

This review examined the effect of communication technologies in diabetes care for the adolescent and young adult population.

#### Outcome

All outcomes were included.

### Data extraction strategy

Two reviewers (PS and SM) performed a systematic screening of identified evidence to determine eligibility, apply quality criteria, and extract data from included studies. Due to the wealth of potentially relevant literature, a random selection of 20% of papers was screened at title and abstract sifts by both reviewers to assure consistency and to discuss any disagreements. The remaining 80% of papers were then split equally and screened by the two reviewers. All papers included at abstract level were read and screened for inclusion by both reviewers. The two reviewers carefully evaluated the inclusion of each paper based on the inclusion criteria reported and the reasons for exclusion were recorded. The data extraction was carried out by one reviewer (PS) and checked for accuracy by a second reviewer (SM). There was no language restriction and translations were sought when necessary. Six study authors were contacted to clarify missing data, of which, three responded; two met the inclusion criteria [25,26].

### Quality assessment strategy

The Downs and Black [27] checklist was used in favour of other critical appraisal tools (e.g. Critical Appraisal Skills Programme [28]) to assess the quality of papers meeting the inclusion criteria. This 27-item checklist enables an assessment of randomised and non-randomised studies and provides both an overall score for study quality and a profile of scores not only for the quality of reporting, internal validity (bias and confounding) and power, but also for external validity. As a broad range of study designs have been used in this area of healthcare, the use of a single checklist, in contrast to individual checklists for each study design, was considered more appropriate.

### Evidence synthesis

Due to the presence of clinical, methodological and statistical heterogeneity, it was not appropriate to conduct a meta-analysis. The results have therefore been evaluated in a narrative format to provide a detailed summary and comparison of communication technologies across the reported outcome measures.

## Results

### Search results

**Figure 1:**
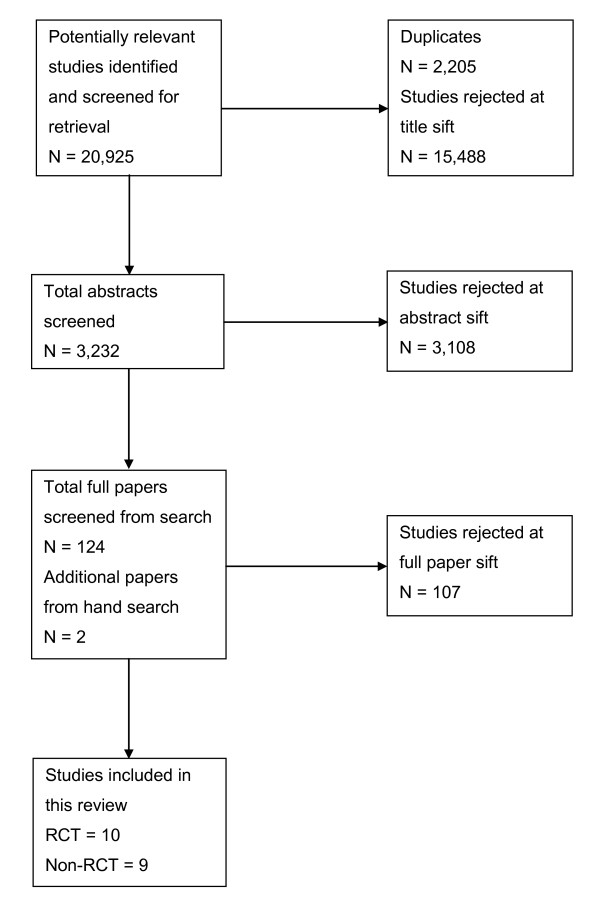
**Summary of study selection and exclusion**.

A flow chart describing the process of identifying relevant literature can be found in Figure 1. Following the removal of duplicates our search identified 18,720 potentially relevant articles. Of the 124 papers identified to read in full 107 were screened and rejected leaving 17 studies included for review. In addition, two additional papers were included from hand and grey literature searches (total n = 19). Three non-English language papers were translated but excluded at full paper sift [29-31]. The reasons for exclusion, at full paper, were: abstract only (n = 11); age (n = 56); non-return of information query (n = 2); inappropriate population (n = 2); no health professional involved (n = 4); not-communication (n = 11); not-diabetes (n = 6); not-primary research (n = 11); and reviews (n = 5).

The 19 included papers (n = 1064 participants) comprised seven study designs: case series [26,32]; case studies [33,34]; observational studies [25,35-37]; qualitative study [38]; randomised crossover trial [39]; and RCT [40-48]. The randomised crossover trial [39] will be treated as a standard RCT in the discussion of findings and all other studies will be termed non-RCTs. A total of five categories of communication technologies were identified from the 19 papers, these include: studies described as using video-conferencing alone or combined with tele-conferencing technologies (n = 2; non-RCT = 2 [25,33]); studies involving mobile telephony, in particular the use of SMS (n = 3; RCT = 2 [39,42], non-RCT = 1 [38]); studies described as telephone support (n = 3; RCT = 2 [44,46], non-RCT = 1 [34]); papers that adopt novel electronic communication devices for transferring clinical information (n = 10; RCT = 6 [40,41,43,45,47,48], non-RCT = 4 [26,32,35,37]); one non-RCT utilised web-based discussion boards [36]. It is important to note that while 19 studies are included in this systematic review there were 18 communication technologies reported; Franklin, 2006 [42] (RCT) and 2008 [38] (non-RCT) report the same intervention.

### Sample characteristics

A total of 978 young people (males = 523, females = 355) with diabetes were included in this review. Study sizes ranged from 5 to 123 (mean = 65.2, SD = 37.2). The mean age was 15.9 (SD = 4.3 yrs).

The RCT studies included a total of 776 young people (Males = 499, Females = 277) with type 1 diabetes. Study sizes ranged from 28 to 123 (Mean = 78.3, SD = 30.78). The mean age of patients ranged from 11.9 to 23.9 years (Mean = 16, SD = 3.74). The non-RCT studies comprised a total of 288 young people with diabetes. Three studies did not report gender distribution, and the participants of the remainder included 94 males and 94 females. Six studies dealt with type 1 diabetes only and two studies with both diabetes type 1 and 2; one study did not identify the nature of the diabetes they researched. Study sizes ranged from 5 to 94 (Mean = 40.8, SD = 32.9). The mean age of patients across the five studies that reported age [33-38] ranged from 10.1 to 22.3 years (Mean = 15.3, SD = 4.3).

### Study characteristics

The RCT studies took place in the UK (n = 3), Australia (n = 2), USA (n = 1), France (n = 1), and Austria (n = 1) Italy (n = 1) and Denmark (n = 1). The majority (n = 7) involved two groups (e.g. intervention and control group), whereas three studies [42,44,47] used an intervention and two comparison groups. The non-RCT studies took place in the UK (n = 1), Australia (n = 1), USA (n = 5), Germany (n = 1), and Italy (n = 1). Only one study [35] involved comparison groups (standard users, non-users and intervention users).

The most common primary outcome measures used were blood glucose and HbA1c levels in both RCT and non-RCT studies. Studies varied in duration from 6 to 18 months, (mean duration for RCT = 9.5 months [range = 6 to 18 months], mean follow-up = 1.6 months [range = 0 to 6 months]; mean duration for non-RCT = 10.9 months [range = 2 weeks to 35 months], mean follow-up = 4.1 months [range = 0 to 25 months]). One non-RCT paper did not report duration of technology use, while three non-RCTs did not report duration of follow-up.

### Quality assessment and evidence synthesis

The Downs and Black overall ratings for the included papers ranged from 2-25 (median = 15; mean = 13.6; SD = 5.9), where low ratings represented poor quality and high ratings represented good quality (Maximum rating of 28). Five papers were rated below 10; eleven between 10 and 20, and three above 20.

All ten RCTs had a clear hypothesis/aim/objective and outcomes as well as clearly defined characteristics of participants. Eight RCTs had clearly described communication technologies of interest [39,41-47] and eight partially described the distributions of principal confounders in each group of participants to be compared [39-43,45-47]. Three non-RCTs were identified as having a clear hypothesis/aim/objective [25,32,35] clear main outcomes [33,35,37], and six non-RCTs clearly described their characteristics of the participants included in the study [32-37].

The following section presents a narrative synthesis of the evidence from all research designs, and includes a description of the communication technologies, the sample and study characteristics, and main findings for the outcomes measured. Full details can be found in Additional File 1.

### Communication technologies

A total of five categories of communication technologies were identified. A description of the studies will be provided in the following section. Further details can be found in Additional File 2.

#### 1. Video- and tele-conferencing

Two studies described as using video-conferencing alone or combined with a tele-conferencing technology [25,33]. One study [25] conducted routine telepaediatric diabetes sessions over video-conferencing software, and aimed to assist young and geographically remote patients to access their regular healthcare providers. The other [33] compared the reporting of blood glucose levels, insulin doses and food intake by telephone, video-phone, and email with the intention of identifying any significant advantages of a particular modality. Again, the technology was targeted at young people who had difficulty in attending the clinic regularly and were unable to maintain glycaemic control.

#### 2. Mobile telephony

Three studies involved the explicit use of mobile telephony, including SMS (text messages) [38,39,42]. Two of these [38,42] report on the same "Sweet Talk" technology. Sweet Talk is a text-messaging support system that is informed by social cognitive theory. It offers the user feedback, information, tips, and reminders with regards to self-management tasks such as insulin injections, blood glucose testing, diet and exercise. The third study [39] used a telemedical support program which collects and processes diabetic condition data, received through SMS and general packet radio service (GPRS), personalised messages with specific diabetes health advice where then sent.

#### 3. Telephone-support

Three studies had a telephone-support based technology [34,44,46]. In one study [46] the technology was based on an experienced paediatric diabetes educator engaging in bimonthly telephone calls. Topics covered insulin, carbohydrate intake, and blood glucose values. In a similar study [44] an experienced paediatric diabetes educator also provided assistance and support using a problem-solving strategy. Calls were used to collect or feedback HbA1c results. Finally, a third study [34] used an intensive, behavioural-health program to effectively communicate with young people who live in geographically remote areas. The technology used target-setting and monitoring to improve patient clinical outcomes.

#### 4. Novel electronic communication

Ten papers described novel electronic communication devices for transferring clinical information [26,32,35,37,40,41,43,45,47,48]; these had been either designed or adapted for the research, or were devices that are not widely commercially available. Most took the form of a synchronised glucometer. Seven studies [32,37,40,43,45,47,48] developed handheld glucose meters with memory storage which could be connected to the PC/internet and at which point recorded clinical data were transmitted. Patients were usually requested to enter clinical and user-defined events which were intended to be transmitted; some such systems allowed automatic collection and transmission of metabolic data from patients to healthcare professionals. Several systems identified incidents of hypoglycaemia and detected high and low glucose levels as well as insulin dose changes, exercise and event marker entries. They incorporated hardware and software innovations; generally the software was capable of downloading, conducting analysis and printing blood glucose management results, which was then analysed by the diabetologist or other health care practitioner. In should be noted that in some instances feedback to the patient could not be provided via a communication technology and was therefore provided in other forms i.e. discussion over telephone [43,48].

Several other communication technologies were reported, for example, a software package which was synchronised with insulin pumps [35]. Data were transmitted through a personal computer and uploaded to a dedicated internet site. Reports could be produced, and feedback was provided by the health provider. No therapeutic recommendations were produced by the software. Other studies have engaged with multiple technologies; web-based education, online communication and remote glucose monitoring [26]. Web-based education involved the recording of time online, pre- and post-test scores, patients were also given access to educational information. In an evaluated system of a glucose self-monitoring, real-time result transmission and feedback were mediated through a diabetes specialist nurse [41]. Patients were provided with graphical phone-based output and nurse-initiated support.

#### 5. Web-based discussion boards

Only one paper [36] utilised web-based discussion boards. The technology was targeted at older adolescents and young adults. The diabetes educator introduced new educational diabetes education material each week. It included goal-setting exercises, personalised feedback, group discussions and role-playing. A majority of users were able to interact with the system without additional training.

### Main findings for outcomes measured

The following section provides a synthesis of all outcome measures reported. We have categorised these into three subheadings.

#### 1. Clinical outcomes

HbA1c and/or blood glucose levels were measured in all RCTs. The mean changes in HbA1c values were calculated at baseline and follow-up (see Table 2). A total of six studies showed a positive improvement in HbA1c following the intervention [40-43,47,48] with four studies reporting detrimental increases in HbA1c levels in one or both intervention groups [44-46]. Two studies found a significant difference in HbA1c between the intervention and a comparison group at the end of the study [42,47]. There was no defining commonality in studies that reported HbA1c increases or decreases (e.g. study quality, type and duration of intervention).

**Table 2:** Mean HbA1c Improvements across groups, study length and quality in Randomised Control Trials

Study	Intervention group Mean			Comparison group 1 Mean			Comparison group 2 Mean			Study length	Quality score
	
	Before	After	**Diff**.	Before	After	**Diff**.	Before	After	**Diff**.	Months	(0-28)
**Text messages**											
Rami 2006 [39]	9.1	9.2	0.1	9.3	8.85	-0.45*				6	12
Franklin 2006 [42]‡‡	10.0	9.2	-0.8*	10.1	10.3	0.2	9.8	10.1	0.3	12	19

**Telephone support**											
Howells 2002 [44]	8.4	8.7†	0.3	8.9	9.5†	0.6*	8.5	8.8*	0.3	12	22
Nunn 2006 [46]	8.15	8.85	0.7*	8.32	8.82	0.5*	-	-		7	20

**Novel electronic communication**											
Chase 2003 [40]	9.0	8.6	-0.4	8.9	8.6	-0.3	-	-		6	15
Farmer 2005 [41]	9.2	8.6	-0.6*	9.3	8.9	-0.4*	-	-		9	25
Cadario 2007 [48]	9.5	8.8	-0.7*	9.1	9.1‡	0	-	-		18	15
Gay 2006 [43]	9.22	9.12	-0.1	9.17	9.27	0.1	-	-		6	18
Marrero 1995 [45]	9.4	10	0.6*	9.9	10.3	0.4*	-	-		12	16
Rosenflack 1993 [47] **	10.3	8.9	-1.4*	9.3	9.3	0	9.8	9.6	-0.2	12	15

‡‡ Significant difference between HbA1c levels for intervention group compared to comparison group 1 (p < .001)

‡ Comparison group was given intervention after 6 months due to the significant decline in HbA1c at 3 and 6 months compared to intervention group (p = .03)

* Significant change between before and after HbA1c level (p < .05)

** Significant difference between groups at end of study (p < .01)

† Approximation based on information provided in tables, true mean HbA1c (%) results not reported

The impact of communication technologies on HbA1c levels were measured in five non-RCTs [32-35,37]. All studies reported improvements in HbA1c; but not all were significant [33]. Telehealth reduced HbA1c levels in the sample of two participants (2.4% and 3.5%) [34]. Similarly, an internet-based insulin pump monitoring system was associated with improved glycaemic control in children with type 1 diabetes [35] and telemedical care reduced the number of hypoglycemias [37]. In a case series of five participants [33], telehealth facilitated the treatment of adolescents with poor glycaemic control, metabolic control was not improved or maintained enough for the adolescents to be free from the risk of complications.

#### 2. Behavioural and psycho-social outcomes

Only one RCT [45] reported clear improvements in quality of life, patient-caregiver interaction, and an increase in the perceived importance of glycaemic control. The same study reported a decrease in perceived mastery in the control group and a decrease in family problem-solving in the intervention group, demonstrating the balances that need to be considered with increased clinician communication. Furthermore, no significance differences were found in communication, roles, affective responsiveness, behaviour control, or general family functioning subscales of the Family Assessment Device (FAD) at baseline or post study. For a similar type of technology [42] patients who received conventional therapy and the 'Sweet Talk' intervention scored better self-efficacy than those using conventional therapy alone. It was also reported that the technology improved patient's perception of quantity of support but had no impact on diabetes knowledge score. In contrast, no significant differences were found in other studies between intervention and control group for self-care behaviours, such as testing overnight, and frequent blood glucose monitoring [35], and sex differences on mean scores for, barriers to adherence, diabetes knowledge, problem solving and rational problem solving [44].

In analysing the content of messages between patients and healthcare provider only 4% of messages contained information on the patients' own diabetes self-management status, with 56% of these containing solely blood glucose values and no participants opted to use date reminders or personal supporter reminders [38].

One case study reported an electronic data collection and transfer device enabled continuity of care, improved access and activities for patients with diabetes [32].

In summary, several technologies appear to offer patients limited improvement in quality of life, continuity of care and access.

#### 3. Self-care and cost outcomes

Two RCTs reported cost reduction [40,48] and improvements in self-care [42,44]. Seven studies noted successful metabolic data transmission [39,40,42,43,45,47,48]. Three RCTs [41,43,45] reported technical problems. Only one study reported that patients were required to be trained in order to utilise the equipment; this training was conducted by the academic researchers [48]. One study reported that no training was required to operate the software as it was widely used in their clinic [45]. Two studies reported improvements in usability and satisfaction of technology [39,43], however one study found no significant difference between a modem transmission of glucose value device and usual face-to-face care (p = .81) [40]. No papers reported outcomes relating to: transparency of care; delivery guidelines; or equity in access to health care.

Patients, families and school nurses in one study who had chosen to transmit blood glucose data and participate in online education expressed satisfaction with the technology, the process and the improved communication [26]. An important technical consideration noted in one study was that most participants connected to the online services between 9pm and 11pm, more frequent use was seen in the first and second months (1,460 and 1,417 hits, respectively) and messages posted on a discussion board may be improved through designated topics being assigned each week [36]. However, one study found no significant reduction in the number of visits to the department and patients requested that a human link with their physician be continued which will require consistent organisational and economic effort [32]. Participant involvement in some studies relied heavily upon reminders and encouragement from diabetes educators and immediate family members. As noted in the RCT evidence, no papers reported outcomes relating to: transparency of care; delivery guidelines; or equity in access to health care.

#### 4. Frequency of contact

Fifteen papers [25,32,35,36,38-48] reported that communication technologies increased the frequency of contact between patient and healthcare professional; although much of this contact was a requirement of the intervention (see Table 3). Two papers reported a significant correlation between improvements in HbA1c and increased contact [35,47], however five papers [38,39,41,42,45] found no support for this relationship.

**Table 3:** Examination of frequency of contact between patient and health care professional in study interventions

Intervention categories and studies	Intervention increases contact between patient and health care professional?	Results based on comparisons between study participants?	Notes
**Video Conference**			
Gelfand, 2003 [33]	Unclear	NA	No adequate discussion
Smith, 2009 [25]	Yes	NA	Increased communication in some participants

**Mobile phone**			
Franklin, 2006 [42]	Yes	Yes	Improved perception of quantity of support but no clinical impact (p > .05)
Franklin, 2008 [38]	Yes	Yes	Found no statistically significant correlation (p > .05)
Rami, 2006 [39]	Yes	Yes*	No adequate discussion

**Telephone**			
Adkins, 2006 [34]	Unclear	NA	No adequate discussion
Howells, 2002 [44]	Yes	Yes	Instructed to increase contact in intervention
Nunn, 2006 [46]	Yes	Yes	Instructed to increase contact in intervention

**Novel communication**			
Cadario, 2007 [48]	Yes	Yes	Intervention design could lead to more clinician contacting patient
Chase, 2003 [40]	Yes	No	Instructed to increase contact in intervention
Corriveau, 2008 [35]	Yes	Yes**	Intervention design could lead to more clinician contacting patient. Fewer face-to-face consultations (2.8 +/- 0.2 (SE) vs. 3.5 +/- 0.1)
D'Annunzio, 2003 [32]	Yes	NA	Intervention design could lead to more clinician contacting patient
Farmer, 2005 [41]	Yes	Yes	Real-time data transfer and design of study may increase communication. No significant influence with contact frequency and change in HbA1c (p = .6)
Gay, 2006 [43]	Yes	Yes	Instructed to increase contact in intervention
Liesenfeld, 2000 [37]	Unclear	NA	Descriptive statistics only. No adequate discussion
Malasanos, 2005 [26]	Unclear	NA	No formal presentation of results
Marrero, 1995 [45]	Yes	Yes	Intervention design could lead to more clinician contacting patient. No significant relationship with communication
Rosenflack, 1993 [47]	Yes	Yes†	Intervention 338 [255-490]. Comparison A 231 [160-350] (p < .007). Comparison B 96 [50-150] (p < .0001) ‡

**Web-based**			
Gerber, 2007 [36]	Yes	NA	Increased contact during initial stages of research then decline

* Comparison between low and high message senders (+/-50%); ** Comparison between rural and urban patient visits;

† 'Time consumption' compared between intervention and 2 control groups; ‡ Value based on sum of minutes per patient per year

## Discussion

Treating diabetes in adolescents and young adults is a challenge for patients, parents and medical practitioners, and poor glycaemic control is common [49]. The current systematic review is the first to evaluate the impact of communication technologies between young patients with diabetes and health care professionals. We found some evidence of the impact of communication technologies on relaying information; enabling informed decision making; promoting 'better' health behaviours; and improving the frequency of contact between patient and healthcare professional. However, there is substantial variation in the quality of the papers and caution is needed when interpreting these findings. It is unclear which forms of communication technologies are most effective due to the inconsistencies in the reported findings and difficulties in categorising technologies to allow reliable comparison. Furthermore, there is uncertainty about the effect of age on the outcomes due to the lack of subgroups analyses on the broad age ranges included in the studies. The use of such communication technologies for improving the frequency of contact for conditions that require close monitoring, clinical assessment and early intervention to avoid adverse events such as hospitalization or emergency visits should be researched further. The higher quality study [41] incorporating several novel technologies and communication modalities provides a model study design.

From the identified studies, there is evidence that young people with diabetes appear to derive some benefits from communication technologies, but despite this, their use in clinical practice is still tentative [50]. Before widespread introduction of communication technologies into everyday practice to complement communication, treatment and management, more research on the effectiveness, cost effectiveness, acceptability and adoption by health professionals is needed. Furthermore, since none of the studies included were aimed at or involved parents of young people, research might consider the attitudes of young people towards the involvement of their parents or carers in communication technologies.

The present review provides some support for previous claims [15] that a combination of enhanced self-efficacy with motivation and knowledge may enable adolescents and young adults to change their health behaviours, which in turn, may change their clinical outcomes (e.g. HbA1c). It is important to recognise, however, that the purpose and function of contact was often different across these broad heterogeneous interventions. Further research exploring the impact of increased communication contact on clinical outcomes is needed.

In focusing first on the RCT evidence, many forms of communication technologies were shown to lower HbA1c levels; but not always. The question remains whether these systems can become substitutes for the face-to-face visits in specific patients. It is important to note that many studies focused on patients who had poor glycaemic control at the outset - hence the findings in such studies might only relate to this sub-population, and not to the wider population of young people who have reasonably well controlled diabetes, this needs to be investigated further.

### Limitations

While many of the papers reviewed were statistically and methodologically robust, there was still a wide range in Downs and Black scores. This highlights a short-fall of large-scale, high-quality empirical research conducted in this area. Several studies reported high or unclear dropout and withdrawal rates. Other concerns surround the generalisability of findings from the included studies; the representativeness of patients, providers and practices in these studies is likely to be variable. In particular, the variable study durations (6-18 months) could have influenced the glycemic data reported across the studies included. The majority of the non-RCT studies were located in the US, whereas more RCTs took place in the UK.

## Conclusions

The present study provides limited evidence that some communication technologies may significantly and positively affect clinical outcomes in young patient populations with diabetes. Further research is needed to explore the generalisability and applicability of such findings, costs, and acceptability and satisfaction issues for patients and healthcare professionals. Future studies should provide clearer reporting of the training needs of patients and providers to master the equipment and the implications of implementing technology in diabetes care. It is also important to investigate the various impact of changing the tasks and duties of the diabetes team as the new technology is implemented (e.g. time tradeoffs), increase physicians' workload due to the learning process required by the new tool, assistance to patients for technical support, need for organisational and clinical protocol changes. Further considerations for health professionals relate to the potential changes to work patterns, services, roles, legislation, and reward mechanisms to help healthcare professionals use these technologies efficiently to communicate with their patients [51].

Unlike some of the reported adult studies in this area (see [52] for a review of teleconsultation in diabetes care) only four of the included studies provided minimal discussion of the attitudes of young people and health professionals towards the communication technologies.

The present review highlighted the lack of research with young people with diabetes and their health professionals involving communication through social networking sites (e.g. Facebook, Myspace). These forms of communication represent a novel opportunity to improve and engage young people in their health care delivery and to be potentially guided by young people themselves. Furthermore, there was limited or no research involving mobile telephony and VoIP system (e.g. Skype); this represents an opportunity for further research given the increasing use of these types of communication technologies among young people.

The included studies suggest different types of communication technologies have the potential to be practical, cost-effective, and reliable methods of health professionals communicating effectively with young patients with diabetes. However, the breadth of study design, age range and types of communication technologies reported makes the magnitude of benefit and implications for the organization of daily practice difficult to determine. The review highlights that high quality evidence is lacking to support more widespread implementation of communication technologies at this stage and the associated improvements in frequency of contact, on clinical outcomes, remains unclear.

## Competing interests

The authors declare that they have no competing interests.

## Authors' contributions

PS and SM conceived the study. All team members were involved in developing the protocol. JP and JS supervised the study. SM helped design and implemented the search strategies. PS and SM extracted data and synthesised the effectiveness evidence. All authors helped draft and edit the manuscript and have read and approved the final manuscript.

## Pre-publication history

The pre-publication history for this paper can be accessed here:

http://www.biomedcentral.com/1472-6823/11/1/prepub

## Supplementary Material

Additional file 1 **Quality assessment for included papers**. Provides details of the Quality Checklist for Health Care Intervention Studies (Downs & Black, 1998) [27]. Click here for file

Additional file 2 **Main characteristics of the included publications**. Provides details of the characteristics of the reviewed studies. Click here for file
