# Supplementary material for: Systematic review of communication technologies to promote access and engagement of young people with diabetes into healthcare
Source: BMC Endocr Disord. 2011 Jan 6;11:1. doi: 10.1186/1472-6823-11-1 (PMC3024230; doi:10.1186/1472-6823-11-1)
Supplement: Additional file 1 — Quality assessment for included papers. Provides details of the Quality Checklist for Health Care Intervention Studies (Downs & Black, 1998) [27]. [file 1472-6823-11-1-S1.DOC]

**Addition information 1**

**Quality assessment for included papers: Quality Checklist for Health Care Intervention Studies (Downs & Black, 1998) [27]**

| **Randomised Controlled Trials** | | | | | | | | | | | | | | | | | | | | | | | | | | | | |
| --- | --- | --- | --- | --- | --- | --- | --- | --- | --- | --- | --- | --- | --- | --- | --- | --- | --- | --- | --- | --- | --- | --- | --- | --- | --- | --- | --- | --- |
| First author year | Q1 | Q2 | Q3 | Q4 | Q5 | Q6 | Q7 | Q8 | Q9 | Q10 | Q11 | Q12 | Q13 | Q14 | Q15 | Q16 | Q17 | Q18 | Q19 | Q20 | Q21 | Q22 | Q23 | Q24 | Q25 | Q26 | Q27 | **sum** |
| Cadario 2007 [48] | y | y | y | n | n | y | y | y | y | n | ct | ct | ct | n | ct | y | y | y | y | y | y | y | ct | n | ct | y | n | **15** |
| Chase  2003 [40] | y | y | y | n | p | y | y | n | n | y | ct | ct | n | n | n | y | ct | y | y | y | y | y | y | ct | n | y | n | **15** |
| Franklin  2006 [42] | y | y | y | y | p | y | y | y | y | y | n | n | y | n | n | y | ct | y | n | y | ct | y | y | y | ct | y | y | **19** |
| Farmer  2005 [41] | y | y | y | y | p | y | y | n | y | y | y | y | n | y | y | y | y | y | y | y | y | y | y | y | y | y | y | **25** |
| Gay  2006 [43] | y | y | y | y | p | y | y | n | y | y | ct | ct | ct | n | n | y | y | y | n | y | y | n | y | y | ct | y | y | **18** |
| Howells  2002 [44] | y | y | y | y | n | y | y | n | y | y | y | y | y | ct | y | y | y | y | y | y | n | ct | y | y | y | y | y | **22** |
| Marrero  1995 [45] | y | y | y | y | p | y | y | y | n | y | ct | ct | ct | ct | ct | y | y | y | y | y | y | ct | y | ct | n | n | n | **16** |
| Nunn  2006 [46] | y | y | y | y | p | y | y | n | y | y | n | n | y | n | y | y | n | y | y | y | y | y | y | ct | ct | y | y | **20** |
| Rami  2006 [39] | y | y | y | y | p | y | n | y | y | n | ct | ct | ct | ct | ct | y | ct | y | y | y | ct | ct | ct | ct | ct | ct | n | **12** |
| Rosenfalck 1993 [47] | y | y | y | y | p | y | n | n | y | y | ct | ct | y | n | n | y | ct | y | y | y | y | ct | y | ct | ct | y | n | **15** |
| **Non-Randomised Controlled Trials** | | | | | | | | | | | | | | | | | | | | | | | | | | | | |
| Adkins  2006 [34] | n | n | y | n | n | y | n | n | y | n | ct | ct | ct | n | n | ct | ct | y | y | y | ct | ct | n | n | ct | y | n | **7** |
| Corriveau 2008 [35] | y | y | y | y | p | y | y | n | y | y | n | ct | n | n | n | y | y | y | n | y | y | y | n | n | ct | y | n | **16** |
| d'Annunzio, 2003 [32] | y | n | y | y | p | y | n | n | n | y | ct | ct | ct | n | n | y | n | y | y | y | y | ct | n | n | ct | n | n | **11** |
| Franklin  2008 [38] | n | n | n | y | n | y | n | n | n | y | ct | ct | y | n | n | y | y | y | n | y | y | y | n | n | ct | y | n | **11** |
| Gelfand  2003 [33] | n | y | y | y | n | y | n | n | y | y | ct | ct | ct | n | n | y | n | ct | n | y | y | n | n | n | n | n | n | **9** |
| Gerber  2007 [36] | n | n | y | y | n | y | n | n | y | n | n | ct | ct | n | n | y | n | y | n | y | n | ct | n | n | n | y | n | **8** |
| Liesenfeld  2000 [37] | n | y | y | y | p | y | n | y | y | y | n | ct | ct | ct | n | y | n | y | n | y | ct | ct | n | n | ct | y | n | **12** |
| Malasanos 2005 [26] | n | n | n | y | n | y | n | n | n | n | ct | ct | ct | n | n | ct | n | ct | n | ct | ct | ct | n | n | ct | ct | n | **2** |
| Smith  2003 [25] | y | n | n | n | n | y | n | n | n | n | ct | ct | y | ct | ct | y | ct | ct | ct | y | ct | n | n | n | ct | ct | n | **5** |

**Overall summary across RCTs and Non-RCTs (number of “yes” responses for each question)**

Q1. Clear hypothesis/aim/objective clearly described (n=13); Q2. Main outcomes to be measured clearly described in the Introduction or Methods section (n=13); Q3. Characteristics of the patients included in the study clearly described (n=16); Q4. Interventions of interest clearly described (n=15); Q5. Distributions of principal confounders in each group of subjects to be compared clearly described (n=10)*; Q6. Main findings of the study clearly described (n=19); Q7. Estimates of the random variability in the data for the main outcomes (n=10); Q8. All important adverse events that may be a consequence of the intervention reported (n=6); Q9. Characteristics of patients lost to follow-up described (n=13); Q10. Actual probability values reported for the main outcomes except where the probability value is less than .001 (n=13); Q11. Asked a representative sample of the population to undertake the study (n=2); Q12. Subjects who were prepared to participate who were representative of the entire population from which they were recruited (n=2); Q13. The staff, places, and facilities where the patients were treated, representative of the treatment the majority of patients receive (n=6); Q14. Were identified as attempting to blind study subjects to the intervention they have received (n=1); Q15. Made an attempt to blind those measuring the main outcomes of the intervention (n=3); Q16. Made clear any results that were based on “data dredging” (n=17); Q17. Adjusted for different lengths of follow-up of patients, or in case-control studies the same time period between the intervention and outcome (n=7); Q18. Were identified as using appropriate statistical tests used to assess the main outcomes (n=16); Q19. Reliable compliance with the intervention/s (n=10); Q20. Accurate main outcome measures (n=118); Q21. Patients in different intervention groups (trials and cohort studies), or cases and controls (case-control studies), recruited from the same population (n=11); Q22. Study subjects in different intervention groups (trials and cohort studies,) or cases and controls (case-control studies), recruited over the same period of time (n=7); Q23. Study subjects randomised to intervention groups (n=8); Q24. Randomised intervention assignment concealed from both patients and health care staff until recruitment was complete and irrevocable (n=4); Q25. Adequate adjustment for confounding in the analyses from the main findings (n= 2)

Q26. Losses of patients to follow-up taken into account (n=13); Q27. Sufficient power was described to detect a clinically important effect where the probability value for a difference being due to chance is less than 5% (n=5)

**Key:** y=Yes, n=No, p=Partially, ct=Can’t tell, *=number of “partially” responses
