# Supplementary material for: Systematic review of communication technologies to promote access and engagement of young people with diabetes into healthcare
Source: BMC Endocr Disord. 2011 Jan 6;11:1. doi: 10.1186/1472-6823-11-1 (PMC3024230; doi:10.1186/1472-6823-11-1)
Supplement: Additional file 2 — Main characteristics of the included publications. Provides details of the characteristics of the reviewed studies. [file 1472-6823-11-1-S2.DOC]

**Additional information 2**

**Data extraction of included studies – RCTs**

| **First author, date of publication, country** | **Study design** | **Population** | **Intervention** | **Outcomes** | **Main results** |
| --- | --- | --- | --- | --- | --- |
| Cadario  2007 [48]  Italy | Randomised controlled trial  **Aim:** Evaluate teenagers with T1DM using the Glucobeeb (Gb) | **Total N:** 26  **Mean age (SD):** Int.=14.8 (no SD; R=10.5-20); Con.=14.7 (no SD; R=10-19.8)  **Sex:** M=14; F=14  **Withdrawals:** 0  **Dropouts:** 0  **Recruitment area:** Paediatric Clinic  **Diabetes type:** T1DM  **Further details:** Diabetes duration 2-15 Yrs. | **Technologies used:** Telecare (Glucobeeb)  **No. of study groups:** 2  **Intervention group:** N=14; Glucometer transmission to diabetologist with feedback. HbA1c tested four times per day, data transmitted every two Wks; clinician feedback  **Comparison groups:** N=12; telephone or face to face visits | **Behavioural:** None  **Clinical:** HbA1c% levels  Frequency of side effects & costs  **Care coordination:** None  **Frequency of contact:** Clinician contacting patient | **Average HbA1c% baseline, 3, 6, 18 Mnths.:**  Int=9.5 +/- 2.0, 9.0 +/- 1.6, 9.1 +/- 1.6, 8.8 +/- 2.1; Con=9.1 +/-1.5, 9.4 +/- 1.5, 9.4 +/- 1.5, 9.1 +/- 1.5; HbA1c endpoint Sig. decreased compared to introduction of Gb (p=.01)  **Frequency of contact:** Intervention design could lead to more clinician contacting patient |
| Chase  2003 [40]  USA | Randomised controlled trial  **Aim:** The aim was to evaluate whether modem technology provides effective management of TIDM when used in lieu of a clinic visit | **Total N:** 70  **Mean age (SD):** Int.=17.4 (1.7); Con.=17.2 (1.5)  **Sex:** M=30; F=33  **Withdrawals:** 7  **Dropouts:** 0  **Recruitment area:** Pediatric & young adult diabetes centre  **Diabetes type:** TIDM  **Further details:** Pts. took at least 2 insulin injection per day | **Technologies used:** Modem transmission of glucose values  **Number of study groups:** 2  **Intervention group**: N=30; Modem group clinic visits at 0 & 6 Mnths., electronic glucose transmission every two Wks  **Comparison groups:** N=33; clinic visits at 0, 3, & 6 Mnths. | **Behavioural:** Degree of glycaemic control;  **Clinical:** Incidence of acute diabetes complications  **Care coordination:** Cost of care, Pt. satisfaction  **Frequency of contact:** Clinician contacting patient | **Mean baseline GHbA1c (p=0.89):** Int.=9.0 +/- 1.2; Con.=8.9 +/- 1.1  **GHbA1c values at 6 Mnth (p=0.96):** Int=8.6 +/- 1.7; Con=8.6 +/- 1.2  **Cost of care**  Sig. Diff. in cost between groups (p<.001). Avr. time spent per clinic visits: 95 Min. (60-128); Avr. cost of clinic visit was $246 (235-310); Training was $35.00  **Pt. satisfaction:** High satisfaction with care. No Sig. Diff. between groups (p=0.81).  **Incidence of acute diabetes complications**  No Sig. Diff. between groups on diabetic ketoacidosis & hypoglycaemia events  **Frequency of contact:** Intervention design could lead to more clinician contacting patient. Instructed to increase contact in intervention |
| Farmer  2005 [41]  UK | Randomised controlled trial  **Aim:** Evaluate whether a system of telemedicine support can improve glycaemic control in T1DM | **Total N:** 93  **Mean age (SD):** Con=23.2 (4.2); Int=24.5 (4.2)  **Sex:** M=55; F=38  **Withdrawals:** 6  **Dropouts:** 6  **Recruitment area:** Young adult clinic  **Diabetes type:** T1DM  **Further details:** Suboptimal or poor glycaemic control with a lower A1c limit of >=8.0% & a upper limit A1c limit of 11.0% for the last 2 results  Mean diabetes duration=12.1 (6.7) Yrs. Note that all Pts. aged 18–30 Yrs. | **Technologies used:** Real-time telemedicine support for glycaemic control  **Number of study groups:** 2  **Intervention group:** N=47;  Clinical advice & structured counseling from diabetes specialist nurse (DSN) in response to real-time blood glucose (BG) test results  **Comparison groups:** N=46;  Results transmitted to server but not available to DSN | **Behavioural:** None  **Clinical:** BG Levels; HbA1c% levels  **Care coordination:** Nurse contact; Technical problems  **Frequency of contact:** Clinician contacting patient | 51,165 BG. results were transmitted. 29,765 Int. & 21,400 Con.  **Blood glucose:** Number of Wks. at least 7 blood tests were taken was Sig. Diff. between groups (27.3 +/- 11.8 & 18.8 +/- 11.1, Diff. 8.4 [95% CI 3.7-13.1], p=<.001). During week 36 of trial median number of readings sent by participants from Int. was 11 (IQR 1-28) compared with 0 (0-7) for Con. (p<.0001). Median BG level for the Int over 9 Mnths. was 8.9 mmol/l (5.4-13.5) vs 10.3 mmol/l (6.5-14.4) for Con. (p=<.001)  **Reduction in A1c BL - 9 Mnths.:** I=9.2 +/- 1.1 to 8.6 +/- 1.4 (Diff. 0.6 [95% CI 0.3-1.0] p=.001). C=9.3 +/- 1.5 to 8.9 +/- 1.4 (Diff. 0.4 [0.03-0.7], p=.04). The Diff. in change between groups was not statistically Sig. at 9 Mnths. (0.2 [-.2 to 0.7], p=0.3. Baseline demographic; clinical characteristics were not associated with subsequent n. of Wks. in which participants tested BG.  **Nurse contact**  601 contacts initiated by nurse to Pts. allocated to Int. (Avr. 13 per Pt., 1 per 2.5 Wks.). Duration was 7 Min. 9s (+/-4 Min., 15 s). No association between total nurse contact time & change in A1c over trial (p=.6)  **Technical problems**  Inability to transmit results due to GPRS (48 occurrences in Int. & 11 in Con.). Other tech Difficulties included cable linking the meter & phone, damage or theft to phones & need to replace batteries. These problems were recorded (n=51 Int.; n=43 Con.)  **Frequency of contact:** Real-time data transfer and design of study may increase communication. No Sig. influence with contact frequency and change in HbA1c (p=0.6) |
| Franklin  2006 [42]  (see also Franklin 2003 [51]; Waller 2006 [53])  UK | Randomised controlled trial  **Aim:** Evaluate Sweet Talk, a text-messaging support system in enhancing self-efficacy, uptake of intensive insulin therapy & improving glycaemic control in paediatric Pts. with T1DM | **Total:** 92  **Mean age (SD)** *Median:* Con.=12.7 (10.5-14.8); Int1.=14.1 (11.7-15.6); Int2.=12.6 (11.2-15.4)  **Sex:** All male  **Withdrawals:** 15  **Dropouts:** 1  **Recruitment area:** Clinics in Tayside, Scotland  **Diabetes type:** T1DM  **Further details:** None | **Technologies used:** Sweet Talk text messaging system  **Number of study groups:** 3  **Intervention group:** 1. Conventional plus sweet talk (N=33); 2. Intensive therapy plus sweet talk (N=31)  **Comparison groups:** Conventional therapy (N=27) | **Behavioural:** Self-efficacy**;** improved self-adherence  **Clinical:** Glycaemic control  **Care coordination:** Complications & weight gain; Health service utilisation  **Frequency of contact:** Clinician contacting patient | **Glycaemic control:** Mean glycaemic control did not change in Pts. remaining on CIT (CIT al1 10.3 +/- 1.7; CIT + Sweet Talk 10.1 +/- 1.7) but improved in Pts. with intensive therapy & Sweet Talk (9.2 +/-2.2; CI-1.9,-0.5; p<.001)  **Self-efficacy:** Pts. on CIT who received sweet talk scored better self-efficacy than those using CIT without sweet talk (CIT al1 56.0 +/-13.7, CIT with Int. 62.1 +/-6.6, CI+2.6,+7.5; p=.003)  **Improved self-adherence:** CIT al1 70.4 +/- 20.0, CIT with Int. 77.2 +/- 16.1, CI +0.34, +17.4; p=.042. Sweet talk improved Pts. Perception of quantity of support but had no impact on diabetes knowledge score  **Complications & weigh gain:**  Non Sig. increases in acute complications or weight gain  **Health service utilization:**  Increased in group using IIT & sweet talk, with a stepwise increase in mean clinical visits during Yr. of study (3.0, 3.3, & 3.9). Statistically Sig. Diff. between those using CIT, IIT plus sweet talk (95% CI +0.1, +0.6, p=.016). Emergency hotline contacts were more frequent in group using IIT with sweet talk than group using CIT with sweet talk (95% CI+3, +44; p=.02). 81% Of those using Sweet talk felt it had helped their diabetes, 90% wanted to continue receiving messages. 97% of Pts. Liked the frequency but 20% complained about receiving same message repeatedly  **Frequency of contact:**  Intervention design could lead to more clinician contacting patient. Improved perception of quantity of support but no clinical impact (p>.05) |
| Gay  2006 [43]  France | Randomised controlled trial  **Aim:** Evaluate the effectiveness & feasibility of reinforced follow-  up via telecare mediated by the local pharmacist in contact with the hospital team to improve glycaemic control in children & adolescents  with T1DM | **Total N:** 100  **Mean age (SD):** Con=13.5 (2.5); Int=13.2 (2.7)  **Sex:** M=61; F=39  **Withdrawals:** No clear discussion  **Dropouts:** 71  **Recruitment area:** Outpatient clinic  **Diabetes type:** T1DM  **Further details:** None | **Technologies used:** Telecare  **Number of study groups:** 2  **Intervention group**: N=36;  Reinforced follow-up  **Comparison groups:** N=35; Usual follow-up | **Behavioural:** None  **Clinical:** HbA1c% levels  **Care coordination:** Diabetologists replies; Failure in data transmission  **Frequency of contact:** Clinician contacting patient | **Diabetologists replies:** Physicians replied to at least 113 (67.7%) faxes, 81 (71.7%) of which being within 5 days after reception. The percentage of responses given to faxes was maximal (81.3%) at the third Mnth of study, & decreased to 50% by the last Mnth  **HbA1c:** 71/100 children went to a doctor’s appointment at 6 +/- 1 Mnths.: 36 in the RFG & 35 in the UFG. 29 others attended either earlier or later & could not be analysed. Their characteristics did not Diff. from those 71 analysed. No Sig. Diff. between the 2 groups’ 6 Mnth HbA1c level (RFG=9.12 +/- 1.46 vs. UFG 9.27 +/- 1.20, p=.41). No Sig. within-group Diff. between initiation & completion of study: Avr. Diff. HbA1c levels was -0.10 +/- 1.10 in the RFG vs. 0.10 +/- 1.05 in UFG (p=.59 & p=.58, respectively)  **Failure in data transmission:**  100% of pharmacies had windows in successful transmissions vs. 36.4% when transmission failed, p=<.0001. Hospital should have received 550 faxes from 50 children in the RFG: 406 from pharmacists & 144 from families. Only 167 (30.4%) were actually received, 135 (33.3%) from pharmacists & 32 (32%) from families  **Frequency of contact:** Intervention design could lead to more clinician contacting patient. Instructed to increase contact in intervention |
| Howells  2002 [44]  UK | Randomised controlled trial  **Aim:** Evaluate changes in self-efficacy for self-management in young people  with T1DM participating in a ‘Negotiated Telephone Support’ (NTS)  intervention | **Total N:** 79  **Mean age (SD):** C=16.9;  I1= 16.1; I2=16.3  **Sex**: M=39; F=40  **Withdrawals:** 12  **Dropouts:** None described  **Recruitment area:** Clinic  **Diabetes type:** T1DM  **Further details:** None | **Technologies used:** Telephone support  **Number of study groups:** 3  **Intervention group:** 1. Continued routine management with NTS (N=31)**;** 2. Yearly clinical review & 3-Mnthly management of HbA1c with NTS (N=29)  **Comparison groups:** N=31; Continued routine management | **Behavioural:** Self-efficacy; Barriers to adherence; Problem solving; Diabetes knowledge  **Clinical:** HbA1c% levels; BMI  **Care coordination:** None  **Frequency of contact:** Clinician contacting patient | **Barriers to adherence, Problem solving, Diabetes knowledge:** No Diff. between male & female mean scores for SED (170.0; SD=17.3; 127-201), barriers to adherence (89; SD=24.5; 47-150), diabetes knowledge (10.1; SD=2.5; 3-15), problem solving (64.4; SD=13.7; 28-89) & rational problem solving (9.8; SD=4.0; 0-19). Sig. negative correlations found between HbA1c, problem solving & SED. No Sig. correlations found in other measures  **Self-efficacy:** Increase in combined I compared with C (t=1.74; df=76; p=.035. Variation in the psychological measures was independent of age & gender. Over Yr. mean Hba1c increased Sig. (8.6-9.0; p=<.01). Deterioration observed in all groups.  Only Sig. effect was changes in perception of barriers to insulin adherence (beta=0.17), which predicted HbA1c at time 2 (r^2^=77; f=5.64; p<.001)  **Frequency of contact:** Intervention design could lead to more clinician contacting patient. Instructed to increase contact in intervention |
| Marrero  1995 [45]  USA | Randomised controlled trial  **Aim:** Evaluate the efficacy of a telecommunication system to assist outpatient management of pediatric Pts. | **Total N:** 106  **Mean age (SD):** Con=13.3 (4.9); Int=13.3 (4.5)  **Sex:** M=63; F=43  **Withdrawals:** None described  **Dropouts**: None described  **Recruitment area:** Pediatric diabetes clinic  **Diabetes type:** Insulin-dependent diabetes mellitus  **Further details:** None | **Technologies used****:** Telecommunication  **Number of study groups:** 2  **Intervention group:** N=52;  Computer-linked outpatient clinic in which Pts. transmitted self-monitoring of blood glucose from homes to hospital every two Wks.  **Comparison group:** N=54; Standard care | **Behavioural:** Self-esteem; Dependency; Body image; Depression  **Clinical:** Metabolic control  **Care coordination****:** System reliability  **Frequency of contact:** Clinician contacting patient | **System reliability:** TDM system was reliable with less than 1% transmission error  **Metabolic control:** No Sig. between-group Diff. in metabolic control, levels in both groups increased over time (p=.001). No Sig. Diff. in hospitalizations (p=.787) or emergency room visits (p=.614) between groups. No Sig. between- or within-group Diff. for self-esteem, dependency, body image, depression, or need for affection subscales of the OFFER questionnaire. Some Sig. within-group changes observed in coping, mastery, & importance of control subscales. Con. group showed trend toward increased coping (p=.06) & decrease in mastery (p=.06). Both groups exhibited increase over time in importance of Control (p=.01). No Sig. between- or within-group Diff. were found on communication, roles, affective responsiveness, behaviour control, or general family functioning subscales of the FAD at baseline or post study. Sig. interaction & time effect on problem solving & affective involvement subscales.  Int. decreased in problem solving scores, while both groups had small but Sig. decrease in affective involvement scores (p=.03). Diabetes-specific quality of life showed no between- or within-group Diff. over course of study. No between- or within-group Diff. on 2 dimensions of parent-child responsibility: diabetes regimen & special presentation activities. Sig. increase reported by both groups over time for activities related to general health, indicating that children were assuming more responsibility. No Sig. between-group Diff., at conclusion of study, for use of SMBG data, importance of self-monitoring, or anxiety concerning physician or nurse review of the records. Int. had fewer negative perceptions at 1yr. concerning necessity of keeping records & ‘sticking their fingers’ (p=.001).  Pts. in the Int. initiated twice as many calls to nursing staff to discuss diabetes control, regimen adjustments, & general diabetes-related concerns as Pts. in Con. group (p<.001)  **Frequency of contact:** Intervention design could lead to more clinician contacting patient. No Sig. relationship with communication |
| Nunn  2006 [46]  Australia | Randomised controlled trial  **Aim:** Evaluate if scheduled telephone calls from a pediatric diabetes educator to children who have T1DM improve HbA1c level | **Total N:** 123  **Mean age (SD):** Con=11.9 (3.0); Int=11.9 (3.7)  **Sex:** M=69; F=54  **Withdrawals** 7  **Dropouts:** 16  **Recruitment area:** Hospital  **Diabetes type:** T1DM  **Further details:** Had T1DM for more than a Yr. & had achieved poor control with HbA1c level >8% | **Technologies used:** Telephone support  **Number of study groups:** 2  **Intervention group:** N=60;  Normal care & bimonthly telephone support  **Comparison groups:** N=63; Normal care | **Behavioural:** Strengths &  Difficulties Questionnaire (SDQ); Indicators of Social & Family Functioning (ISAFF); Difference in the rate of hospitalization; Modified version of the Test of Diabetes Knowledge (mTDK); Change in mTDK per cent correct from the first to the last visit  **Clinical:** HbA1c% levels  **Care coordination:** Cost of service  **Frequency of contact:** Clinician contacting patient | **HbA1c:** Sig. increase in mean Hba1c in all Pts. Mean at first visit was 8.24% (SD=0.10) & final visit 8.84 (SD=0.11); p<.001. In Con. group mean HbA1c increased from 8.32 to 8.82 & Int. group from 8.15 to 8.85 (p=.24). For 19 subjects in Con. group & 20 in contact group, discussions were held with parents (98% mothers) as well as children. For these children HbA1c in Con. group increased 0.46% (SD 0.43) & in phone group 0.92 (SD=0.99; p=.07). Those without parental involvement HbA1c in Con. increased 0.53 (SD=1.03) & phone 0.60 (SD=0.98; p=.74). No improvement in HbA1c after adjustment for age & gender. Review of data from May 2000 to December 2003 indicates that over Yr., the Mnth mean HbA1c level at time of concealment was 8.4% & 210 days later 8.58%  **Hospitalisation**  Imbalance in hospital admissions (standardized to rate per Yr.) between Con. (0.4 per Yr. & 1.0 d/Yr.) & Int. (1 per Yr. & 3.0 d/Yr.) in 270 days before each individual entered study.  Diff. was maintained tHrough observation with rates in Con. (0.6 per Yr. & 1.2 d/Yr.), Int. (1.0 per Yr. & 2.6 d/Yr.). Change was not Sig. (admission, p=.57 & admission days, p=.50). No statistical Diff. between mTDK change in percentage of correct answers for either Pts. or parents. No Sig. Diff. between SDQ sub-scores of 2 groups. Self-recorded SDQ scores were consistent with parent ratings. No Diff. in measures of compliance. In follow-up SDQ parents were asked in involvement in study was helpful. (0, no help; 1, a little; 2, medium amount; 3, a great deal). Those in phone group reported greater benefit. Scores 0, 1, 2, & 3 were 7%, 36%, 43%, 14% in Int., compared with 34%, 38%, 24% & 4% in Con. group (p=.003). This response was not associated with the observed change in HbA1c level  **Cost of service**  Con. subjects families initiated phone calls to the team at a rate of 1.1 calls per Yr. & those in Int. group 0.7 calls per Yr. 782 calls were made over period – mean calls were made between 1600 & 2130 Hrs. Cost of $A3950 per Mnth was spent to maintain contact with Int. group. Coefficient of variation for DCA 2000 HbA1c level was 4.9%  **Frequency of contact:** Intervention design could lead to more clinician contacting patient. Instructed to increase contact in intervention |
| Rami  2006 [39]  Austria | Randomised crossover trial  **Aim:** Evaluate the  feasibility of a telemedical support program & its  effect on glycaemic control in adolescents with T1DM | **Total N:** 36  **Mean age (SD):** Mean & SD not reported; Median (range) at start; Overall = 15.3 (10.7–19.3)**;** Int1.=14.5 (12.9–19.3)  Int2.=16.2 (10.7–19.0)  **Sex:** M=20; F=16  **Withdrawals:** None described  **Dropouts:** None described  **Recruitment area:** None described  **Diabetes type:** T1DM  **Further details:** None | **Technologies used:** Telemedical support program  **Number of study groups:** 2  **Intervention group:** 1 group started using the telemedicine support system & weekly advice for 3 Mnths. & then switched to routine scheme with a daily writing protocol (paper diary, PD) & a clinical visit after 3 Mnths.  (TM-PD group, N=18), while the other group started with their PD & switched thereafter to TM (PD-TM group, N=18)  **Comparison groups:** None | **Behavioural:** None  **Clinical****:** Glycaemic control  **Care coordination:** Pt. satisfaction  **Frequency of contact:** Clinician contacting patient | **Glycaemic control:** GC improved during the TM phase, while deteriorated during PD phase: TM-PD group HbA1c (median=9.05, 8-11.3) at baseline, (median=8.9, 6.9-11.3) at 3 Mnth, & (median 9.2, 7.4-12.6) at 6 Mnth, & PD-TM group (median 8.9, 8.3-11.6) at baseline, (median 9.9, 8.1-11) at 3 Mnth, (8.85, 7.3-11.7) at 6 Mnth, p<.05. 9 Pts. (25%) sent only <50% of required 4 daily BG values. Comparing metabolic control of those <50 + >50 no Sig. Diff. No severe hypoglyceamia, 2 events of diabetic ketoacidosis were observed, 1 during TM phase 1 PD phase. BMI (kg/m^2^) & insulin dosage (IE/kg/d) were not Diff. between 2 groups, no influence of age, sex or social background on metabolic control. During TM phase GPRS was not available, & network capacity overload/connection problems were reported several times by 26/36 Pts. resulting in error messages. Study reports positive results in Pt. satisfaction  **Frequency of contact:**  Intervention design could lead to more clinician contacting patient. Instructed to increase contact in intervention |
| Rosenfalck  1993 [47]  Denmark | Randomised controlled trial  **Aim:** Evaluate the clinical efficiency of a computer-based registration & analysis system (Diva System), designed to support diabetes care | **Total N:** 56  **Mean age (SD):** Con1.=16.6 (No SD, range 14.5-20.2); Con2. =17.9 (No SD, range 14.3-20.7); Int.=16.9 (No SD, range 14.3-20.0)  **Sex:** M=56; F=0  **Withdrawals:** 0  **Dropouts:** 0  **Recruitment area:** Hospital  **Diabetes type:**T1DM  **Further details:** Had T1DM for over 2 Yrs. & HbA1c above 8% | **Technologies used:** A computerized diary telecare system.  Pt. can transmit data via telephone.  **Number of study groups:** 3  **Intervention group:** N=9  (Diva group) Diva supported intensified outpatient regimen  **Comparison groups:** 1. N=7  intensified outpatient control;  2. N=40, Conventional outpatient control | **Behavioural:** None  **Clinical:** HbA1c% levels  Number of hypoglycaemic episodes  **Care coordination:** Time consumption in the clinic  **Frequency of contact:** Clinician contacting patient | **HbA1c:** Pts. were followed for 12 Mnths. Diva group used system during first 6 Mnths. In this period the HbA1c decreased Sig. 1.6% p < .001, compared to only a slight decrease in the two Con. groups, 0.3 & 0.4% respectively (p>.05). Int. seems to be a supportive tool, which might assist Pts. to better self care & improve metabolic control. However, use of computer system is time consuming (approximately 30 Min. more time per visit)  **Frequency of contact:** Intervention design could lead to more clinician contacting patient |

**Data extraction of included studies – non-RCTs**

| **First author, date of publication, country** | **Study design, aims** | **Population** | **Intervention** | **Outcome** | **Main results** |
| --- | --- | --- | --- | --- | --- |
| Adkins  2006 [34]  USA | Case studies  **Aim:** Unclear. The study described 2 cases from a telehealth intervention for youth whose diabetes is poorly managed | **Total N:** 2  **Mean age (SD):** 13.5 Yrs.  **Sex:** M=0; F=2  **Withdrawals:** None described  **Dropouts:** None described  **Recruitment area:** Not described  **Diabetes type:** T1DM  **Further details:** Adherence problems | **Technologies used:**  Telecare  **Number of study groups:** 2 Individual cases  **Intervention group:** Telephone contact 3 to 4 times per Wk. by behavioural health specialist  **Comparison groups:** None | **Behavioural:** Barriers to treatment  **Clinical:** HbA1c% levels  **Care coordination:** Lower cost treatment | **HbA1c:** Main findings of this study are unclear. Each case had a drop in HbA1c from 10.3% to 7.9% & from 12.9% to 9.4%. Authors concluded that telephone-based, intensive, behavioral-health interventions may address barriers to treatment access by providing lower cost treatment that is easier to access for youth who do not live near a knowledgeable behavioral-health specialist |
| Corriveau  2008 [35]  USA | Observational study  (Retrospective case controlled study)  **Aim:** Evaluate whether an internet-based insulin pump  monitoring system improved glycaemic control in rural &  urban children treated with insulin pump therapy | **Total N:** 94  **Mean age (SD):** 10.1 (3.3)  **Sex:** M=39; F=55  **Withdrawals:** None described  **Dropouts:** None described  **Recruitment area:**  Presbyterian Medical Group Human Research Review Committee  **Diabetes type:** T1DM  **Further details:** Rural & urban children | **Technologies used:**  Internet-based insulin pump monitoring system (Carelink)  **Number of study groups:** 3  **Intervention group:** (N=33)  Carelink users  **Comparison groups:** 1. No-access to Carelink (N=20); 2. Carelink non-users (N=41) | **Behavioural:** Self-care behaviours – dose adjustment  **Clinical:** HbA1c% levels  **Care coordination:** Annualised clinic visits; Uploading content  **Frequency of contact:** Clinician contacting patient; frequency of face-to-face consultations | **HbA1c:** Rural users [con. 7.9 +/- .2 (SE) vs. Int. 7.4 +/- .2 (SE)]. Rural non-users [9.2 +/- .5 (SE) vs. 9.2 +/- .5 (SE)]. Rural no access [8.1 +/- .3 (SE) vs. 8.0 +/- .4 (SE)], p=.79. No change in urban Pts. before & after carelink use  **Annualised visits:** Rural [2.8 +/- .2 (SE)] vs. urban [3.5 +/- .1 (SE)], p=.003  **Content upload per Mnth:** Rural [2.3 +/- .5 (SE)] vs. Urban [2.1 +/- .3 (SE)], p=.75  **Self-care behaviours:** No-access Pts.=50 vs. 68%. Access Pts.=50 vs 64%. Non users (23%) p=.03 & testing overnight (15%) p=.01. Mean frequency of BG monitoring no access was 5.4 +/- 2.3 (SE), non-users 4.3 +/- 1.9 (SE), users 5.3 +/- 1.5 (SE), p=.13  **Frequency of contact:** Intervention design could lead to more clinician contacting patient. Fewer face-to-face consultations (2.8 +/- 0.2 (SE) vs. 3.5 +/- 0.1) |
| d'Annunzio  2003 [32]  (see also Bellazzi 2002 [54])  Italy | Case series  **Aim:** Evaluate the implementation & validation of a telemedicine service to manage young Pts. with T1DM | **Total N:** 6  **Mean age (SD):** Mean not reported  Range=9.9-15.8  **Sex:** M=4; F=2  **Withdrawals:** 0  **Dropouts:** 1  **Recruitment area:** None described  **Diabetes type:** T1DM  **Further details:** Computer literate Pts. included | **Technologies used:**  Electronic communication devise to allow communication between Pt. & hospital  **Number of study groups:** 1  **Intervention group:** Telemedicine  **Comparison groups:** No comparison group | **Behavioural:** None  **Clinical:** Reduction in BG.; Insulin requirement  **Care coordination:**  Links between Pt. & Medical units  **Frequency of contact:** Clinician contacting patient | **Reduction blood glucose:** 9% mean reduction in BG (from 14.6 mg/dl to 133.9 mg/dl). 11% median reduction (from 158 mg/dl to 141.3 mg/dl). HbA1c was reduced in all Pts.  **Insulin required:** Decreased in all Pts. Sig. in 2 (p=.02 & p=.007)  **Links:** Positive correlation between MU+PU & n. of insulin protocol variations (p=.01). Positive correlation between n. insulin protocol variations & reduction in mean HbA1c (p=.02)  **Frequency of contact:** Intervention increased contact between patient and health care professional. Intervention design could lead to more clinician contacting patient |
| Franklin  2008 [38]  UK | Qualitative study of Pts. from Franklin, 2006  **Aim:** Not clear. The authors evaluated how Pts. with T1DM interact with the Sweet Talk system | **Total N:** 64  **Mean age (SD):** 18.4  **Sex:** Not identified  **Withdrawals:** None described  **Dropouts:** None described  **Recruitment area:** Clinics in Tayside, Scotland  **Diabetes type:** T1DM  **Further details:** Participants were those in the 2 Int. groups stated in Franklin, 2006 | **Technologies used:**  SMS messaging  **Number of study groups:** 1  **Intervention group:** Int. group comprises the Conventional plus sweet talk  & intensive therapy plus sweet talk  **Comparison groups:** None | **Behavioural:** Frequency of Pt. interactions; BG testing; association between messaging & Pt. characteristics; Diabetes questions & information; Personal health administration  **Clinical:** None  **Care coordination:**  Social messaging  Technical messaging; Message errors; Responses to system-generated messages  **Frequency of contact:** Clinician contacting patient | **Frequency of Pt. interactions:** 1180 message sent (range 0-240; Median=6). 5 Pts. contributed 52% (614/1180) of the messages. 2 Boys sent 338/1180 messages (29%)  **Blood glucose testing:** Messages containing BG values accounted for 35% of messages (418/1180). 56% of these (232) submitted BG values al1. 2 boys contributed to 81% of total BG texts sent  **Association between messaging & Pt. characteristics:** No associations between total No. messages & Pts. social, clinical demographics (age, gender, duration of diabetes, insulin regimen, HbA1c, social deprivation (p>.05). Females sent more messages unrelated to diabetes (F=1.53 +/- 2.51; M=.09 +/- 0.30; p=.002). Pts. sending messages received a higher number of personalized responses (r=.521, p=.01)  **Diabetes questions:** Message containing questions related to some aspect of self-management made up 6% (74/1180) of all Messages.  **Diabetes information:** 4% (50/1180) Message s contained info on pts. own diabetes self-management status  **Personal health administration:** 5% (59/1180) contained requests for supplies such as insulin pump consumables, BG meters, & insulin travel authorization letters  **Social messaging:** 6% (75/1180) Messages not directly related to diabetes  **Technical messaging:** 7% (86/1180) were related to technical aspects of system. 55 were related to transmission & cost  **Message errors:** 2% (19/1180) of messages were sent to system in error  **Responses to system-generated messages:** 40% (472/1180) were direct reply to system. System newsletters generate most responses (40%, 190/472), daily scheduled messages (30%, 142/472), personal messages (25%, 118/472), & weekly goal reminder (5%, 22/472)  **Frequency of contact:** Intervention increased contact between patient and health care professional. Found no Sig. correlation (p>.05) |
| Gelfand  2003 [33]  USA | Case studies  **Aim:** Not clear. The authors evaluated individual cases who participated in an intensive outpatient treatment programme designed to improve adherence to their regimen & improve metabolic control | **Total N:** 5  **Mean age (SD):** 14.2 (range=12-16)  **Sex:** M=1; F=4  **Withdrawals:** 0  **Dropouts:** 4  **Recruitment area:** Clinic  **Diabetes type:**  T1DM or T2DM  **Further details:**  Individual cases varied | **Technologies used:**  Telehealth management  **Number of study groups:** 5 people  **Intervention group:** NA  **Comparison groups:** NA | **Behavioural:** None  **Clinical:** HbA1c% levels  **Care coordination:** None | **HbA1c:** Karen BL=9.7, 1 Mnth=8.7, 3 Mnth=8.5; Sarah BL=8.7, 3 Mnth=7.1; Thomas BL=13, 3 Mnth=6.4**;** Helen BL=10.2, 1 Mnth=9.9, 2 Mnth=9.5, 3 Mnth=9.4; Lauren BL=0.2, 3 Mnth=10.4, 6 Mnth=9.7 |
| Gerber  2007 [36]  USA | Observational study  **Aim:** Not clear. The authors evaluated a pilot phase of an internet program to assist individuals who are transitioning to adult-centered medical care | **Total N:** 19  **Mean age (SD):** 22.3  **Sex:** M=7; F=13  **Withdrawals:** None described  **Dropouts:** None described  **Recruitment area:**  Chicago Childhood Diabetes Registry & Clinics  **Diabetes type:** T1DM or T2DM  **Further details:** Avr. age of living with diabetes=12 Yrs. (range=8.1-18.3) | **Technologies used:**  Internet-based interaction  **Number of study groups:** 1  **Intervention group:** Online program with discussion board being managed by a diabetes educator **Comparison groups:** NA | **Behavioural:** Self-management  communication  **Clinical:** None  **Care coordination:**  Use of STYLE; Time spent on tasks  **Frequency of contact:** Clinician contacting patient | **Use of STYLE:** Program assessed 4,445 times. 25.8% of hits between 9 & 11pm. Discussion board received heaviest use of services; readings & postings were recorded a total of 2,256 times over 6 Mnths. (range 4 to 576 per pts; median 57, SD=148.5). Discussion board was primarily utilized by 4 participants. Social (emotional) support was dominant theme, with informal support & exchange of stories of secondary importance  **Time spent on tasks:** Varied by subject. Overall, half of the subjects reported completing the modules within 30 Min., while an additional 30% spent between 30 & 60 Min. on each module. At 6 Mnths. 12 subjects (63%) attended individual or group feedback sessions. Half of the subjects felt the modules were “very easy” or “easy” to complete with remainder describing them as “somewhat hard”. 8 of 12 subjects (67%) reported trouble completing module activities on time of10 due to work, school & family  **Self-management:** 6 of 12 subjects (50%) received encouragement to log into STYLE from family ‘often’ or ‘very often’. 10 subjects (83%) stated they experienced greater control of their diabetes following completion of STYLE modules  **Communication:** Most subjects (10/12, 83%) felt comfortable discussing diabetes with physicians only 3 (25%) stated that STYLE improved communication with providers  **Frequency of contact:** Increased contact during initial stages of research then decline |
| Liesenfeld  2000 [37]  Germany | Observational study  **Aim:** Not clear. The authors evaluated a telecare programme designed to enable children or adolescents & their families to improve diabetes therapy & metabolic control at home | **Total N:** 54  **Mean age (SD):** 13.3  **Sex:** M=43; F=18  **Withdrawals:** 6  **Dropouts:** 1  **Recruitment area:**  Regional Health Insurance diabetes centre  **Diabetes type:** T1DM  **Further details:** Pts. lived more than 400 km from centre | **Technologies used:**  Telemedicine  **Number of study groups:** 1  **Intervention group:** Telecare programme - all Pts. were provided with a hand-held glucose meter & palmtop computer with modem to send daily blood glucose readings  **Comparison groups:** No comparison group | **Behavioural:** None  **Clinical:** Glycaemic control; Intensive insulin therapy  **Care coordination:**  None | **Glycaemic control:** HbA1c had dropped by 0.4% (range -3 to +2.2; n=47; p<.05) at end of program. Reduction in mean BG by 11mg/dL. Rate of hypoglycaemic events was Sig. reduced although proportion of readings below 80 mg/dL remained stable. On Avr. 4 Mnths. was required to achieve optimal glycaemic control  **Intensive insulin therapy:** Multivariate regression analysis of a set of 6 therapeutic advices at onset of program (site of insulin injection, type of bedtime insulin, time of bedtime, BG target, number of injections of intermediate-acting insulin per day & correction factor) did not reveal any Sig. model for HbA1c, mean BG, variability of BG, or frequency of hypoglycemia as dependent variables similar analysis revealed a Sig. model for BG (p=.02, r^2^=0.32) containing BG target value (p=.06 & number of intermediate-acting insulin injections (p=.08) as borderline Sig. variables. Model for variable BG (p=.07, r^2^=0.25) & BG target range (p=.06, r^2^=.26) contained correction factor as influential variable (p=.07 & .04, respectively) |
| Malasanos  2005 [26]  USA | Case series  **Aim:** Not clear. The authors provide details of a system of remote blood glucose monitoring & online education for school personnel, families & providers | **Total N:** 44 Pts.; 6 carers; 6 case-managers;18 school nurses  **Mean age (SD):** None described  **Sex:** None described  **Withdrawals:** None described  **Dropouts:** None described  **Recruitment area:**  School  **Diabetes type:** T1DM  **Further details:** None | **Technologies used:**  Online communication & remote monitoring  **Number of study groups:** 1  **Intervention group:** Pts. with diabetes  caregivers, case managers & school nurses were provided with secure email access, allowing blood glucose & other  data transfer  **Comparison groups:** No comparison group | **Behavioural:** Not reported  **Clinical:** Not reported  **Care coordination:** Not reported | Poor reporting of main findings. 50% of school nurses & 100% of case managers completed educational modules on the FITE Website. Over 90% of Pts. & all school nurses received equipment for transmitting BG data to their computers. Those Pts., families & school nurses who chose to transmit BG data & participate in online education expressed satisfaction with the technology, the process & the improved communication |
| Smith  2003 [25]  Australia | Observational study  **Aim:** Retrospective review of experiences with videoconferencing as a technique for delivering specialist diabetes advice, post-acute Pt. care & educational for Pts. & health professionals | **Total N:** Not reported  **Mean age (SD):** Not reported  **Sex:** Not reported  **Withdrawals:** Not reported  **Dropouts:** Not reported  **Recruitment area:** Not reported  **Diabetes type:** Not reported  **Further details:** Total of 21 routine telepaediatric clinics were conducted via videoconference. 16 of these clinics (76%) were for children with diabetes. The remaining 5 clinics were for children with endocrine conditions | **Technologies used:**  Telemedicine & videoconferencing  **Number of study groups:** Retrospective review of consultations  **Intervention group:** 160 consultations & 10 educational sessions were analysed  **Comparison groups:** No comparison group | **Behavioural:**  Videoconference usage (time spent per session)  **Clinical:** None  **Care coordination:**  None  **Frequency of contact:** Clinician contacting patient | **Usage:** 21 clinics were conducted via video conference; 6teen clinics (76%) were specifically for children with diabetes. Each clinic involved median 6 (2-14 Pts.). Routine videoconference clinics ran for median time of 130 Min. (IQR=60-240). 25 single Pt. consultations were facilitated. 21 of these Pts. either were not normally seen during routine clinic program or were seen routinely but required more regular follow-up to monitor & guide management. Several cases required input of more than 1 RCH medical staff member. Mean duration of single consultation was 45 Min. (IQR=30-60). 10 education sessions were delivered via videoconference to either regional staff or Pts. 3 lectures were presented by RCH consultant each lasting 1 Hr. 3 multi-point lectures were given to a network of diabetes educators; each session lasted up to 90 Min. & was delivered to between 4 & 12 sites. 4 education sessions were coordinated for children from isolated rural areas  **Frequency of contact:** Intervention increased contact between patient and health care professional. Increased communication in some participants |
| **Key:** Int. Intervention group; Con. Control group; Sig. Significant; Diff. Difference(s); Min. Minute(s); Hrs. hour(s); Wk(s). Weeks; Avr. Average; BG. Blood glucose; Pts. Patient(s)/Participant(s); Yr(s). Year(s); Mnths.. Months; T1DM. Type 1 diabetes mellitus; T2DM. Type 2 diabetes mellitus; NA. Not Applicable | | | | | |
